# Small GTPases and Stress Responses of *vvran1* in the Straw Mushroom *Volvariella volvacea*

**DOI:** 10.3390/ijms17091527

**Published:** 2016-09-10

**Authors:** Jun-Jie Yan, Bin Xie, Lei Zhang, Shao-Jie Li, Arend F. van Peer, Ta-Ju Wu, Bing-Zhi Chen, Bao-Gui Xie

**Affiliations:** 1 Mycological Research Center, College of Life Sciences, Fujian Agriculture and Forestry University, Fuzhou 350002, China; junjie017@163.com (J.-J.Y.); 18705043595@163.com (B.X.); zhanglei311540@163.com (L.Z.); arendvanpeer@gmail.com (A.F.v.P.); cbz_2006@163.com (B.-Z.C.); 2 State Key Laboratory of Mycology, Institute of Microbiology, Chinese Academy of Sciences, Beijing 100080, China; lisj@im.ac.cn (S.-J.L.); wutaju@163.com (T.-J.W.)

**Keywords:** edible fungi, small GTPase superfamily, superoxide anion, abiotic stresses, RT-qPCR

## Abstract

Small GTPases play important roles in the growth, development and environmental responses of eukaryotes. Based on the genomic sequence of the straw mushroom *Volvariella volvacea*, 44 small GTPases were identified. A clustering analysis using human small GTPases as the references revealed that *V. volvacea* small GTPases can be grouped into five families: nine are in the Ras family, 10 are in the Rho family, 15 are in the Rab family, one is in the Ran family and nine are in the Arf family. The transcription of *vvran1* was up-regulated upon hydrogen peroxide (H_2_O_2_) stress, and could be repressed by diphenyleneiodonium chloride (DPI), a NADPH oxidase-specific inhibitor. The number of *vvran1* transcripts also increased upon cold stress. Diphenyleneiodonium chloride, but not the superoxide dismutase (SOD) inhibitor diethy dithiocarbamate (DDC), could suppress the up-regulation of *vvran1* gene expression to cold stress. These results combined with the high correlations between gene expression and superoxide anion (O_2_^−^) generation indicated that *vvran1* could be one of the candidate genes in the downstream of O_2_^−^ mediated pathways that are generated by NADPH oxidase under low temperature and oxidative stresses.

## 1. Introduction

Small GTPases, a group of guanine nucleotide binding protein monomers with molecular weights ranging from 20 to 30 kDa, widely exist in eukaryotic cells [1]. Small GTPases (sometimes called Ras superfamily GTPases) can bind to GTP and GDP, switching between the activated (GTP binding) and non-activated (GDP binding) states depending on the binding of different substrates, thereby turning on or off relevant metabolic pathways [2]. The Ras superfamily can be further divided into five families (Ras, Rab, Rho, Ran and Arf) depending on the structures and functions of each constituent [3]. The Ras family is important for cell proliferation, differentiation, apoptosis and the regulation of gene expression [4]; the Rho family is involved in cytoskeleton formation, cell polarity, the cell cycle, the regulation of gene expression and the process of mating in yeast cells [5,6,7]; the Rab and Arf families play important roles in transport across cell membranes [8,9,10,11,12]; and the Ran family regulates the transportation of proteins and RNA molecules at the nuclear pore and is crucial for nuclear assembly, spindle formation and the regulation of mitosis [13,14,15].

To date, fungal small GTPases have been annotated and classified in only a few fungi, including *Coprinopsis cinerea*, *Cryptococcus neoformans*, *Laccaria bicolor*, *Ganoderma lucidum*, *Phanerochaete chrysosporium*, *Ustilago maydis*, *Saccharomyces cerevisiae*, *Schizosaccharomyces pombe* and *Schizophyllum commune* [3,16,17,18,19]. The straw mushroom (*Volvariella volvacea*) is a major cultivated edible fungus in China. The genomes of two *V. volvacea* strains, PYd21 and V23, have been sequenced [20,21]. However, the small GTPases in straw mushroom have not yet been annotated or classified. Considering the importance of small GTPases in the growth, development, differentiation and environmental responses of eukaryotes, it is necessary to classify and explore the small GTPases of *V. volvacea*.

The small GTPases of the Ran family are abundant in eukaryotes [4]. Chinnusamy et al. [22] showed that Ran GTPases play an important role in stress responses in plants. Previous studies have also shown that oxidative stress, heat shock, UV irradiation and other abiotic stresses can cause changes in the expression level and cytoplasmic distribution of human Ran GTPase [23]. Similar phenomena have also been observed in yeast [24]. *V. volvacea* should be cultivated at high temperatures (30–35 °C) and can’t grow at low temperature (<15 °C). At low temperature, the mycelium and sporophore are prone to autolysis [20], making it difficult for cultivation and post-harvest preservation. In this study, the small GTPases of *V. volvacea* were annotated based on the genome sequence of the homokaryotic strain PYd21. Only one small GTPase, VvRan1 of the Ran family, was found in *V. volvacea*. A transcript analysis of *vvran1* gene was studied under low temperature and oxidative stress.

## 2. Results

### 2.1. Identification of V. volvacea Small GTPases

A total of 116 putative small GTPase sequences were obtained from the local BLASTP analysis of 11,534 predicted *V. volvacea* amino acid sequences using human RAS-related protein sequences downloaded from the RASOnD database [25] as the reference. Further domain analysis using Pfam revealed 35 sequences containing the PF00071 domain common to Ras, Rho, Rab and Ran, while nine sequences containing the Arf specific-domain PF00025 were identified. Of these, the amino acid sequences for 11 small GTPases were not complete, and the full lengths of their coding sequences could not be obtained by reads re-assembly. Thus, the full-length coding sequences of these small GTPase were amplified and sequenced by Sanger sequencing, which resulted in the full-length coding DNA sequences of these 11 small GTPase and the prediction of their amino acid sequences. By further comparison of the predicted amino acid sequences with the small GTPase databases, two predicted small GTPase sequences (VvRas3 and VvRab5) had extra segments. These extra segments were considered to result from prediction errors and were therefore removed. All of amino acid sequences of the 44 predicted *V. volvacea* small GTPases were obtained and uploaded to GenBank database, the sequences brief information are shown in Table 1.

### 2.2. Classification of V. volvacea Small GTPases

**Figure 1:**
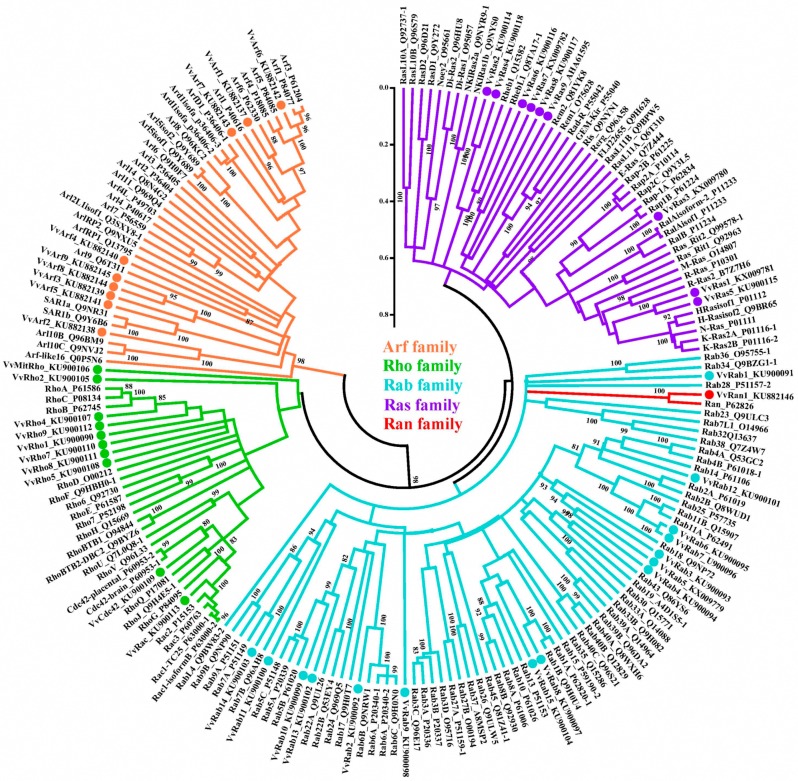
Phylogenetic analysis of human and *V. volvacea* small GTPases. The 151 human small GTPases were cited based on Rojas et al. [3]. The branches were designated as protein name_uniprot ID. The clades of 44 *V. volvacea* small GTPases were labeled with the protein name_GenBank ID. The confidence levels of nodes were tested by bootstrapping 1000 times; scores ≥80% were denoted.

The amino acid sequences of 151 human small GTPases identified by Rojas et al. [3] were downloaded from the RCSB Protein Data Bank [26]. Among these sequences, there were 39 Ras proteins, 22 Rho proteins, 60 Rab proteins, one Ran protein and 29 Arf proteins. The phylogenic analysis of the 44 predicted *V. volvacea* small GTPases using the 151 human small GTPases as references showed that all human and *V. volvacea* small GTPases were clustered into four large clades (Figure 1). The Ran and Rab family proteins were relatively close and were clustered together in the same clade. The 44 predicted *V. volvacea* small GTPases could be divided into the Ras, Rab, Rho, Ran and Arf families. There were one Ras proteins, 10 Rho proteins (of which one, VvMitRho, was a mitochondrial protein), 15 Rab proteins, one Ran protein and nine Arf proteins.

**Figure 2:**
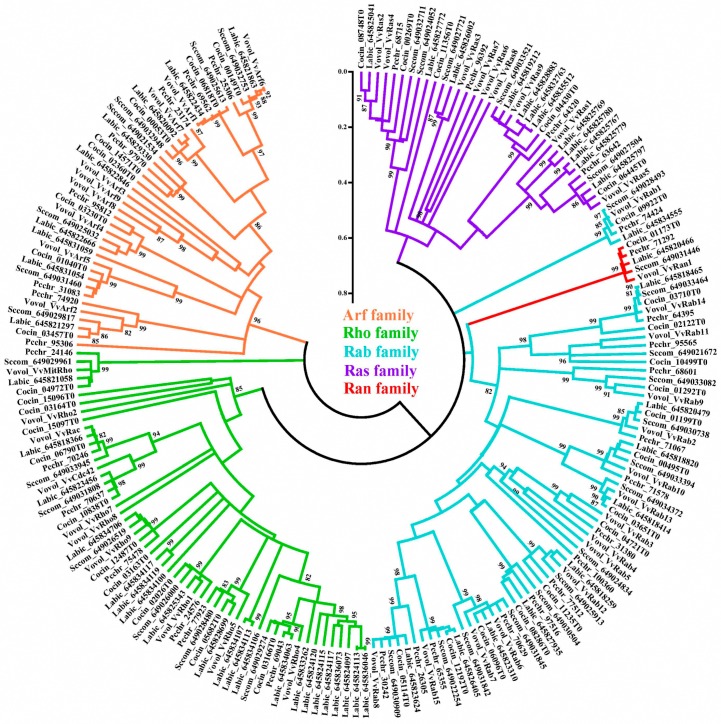
Phylogenetic analysis of small GTPases of 5 basidiomycetes. The clades were labeled with the abbreviated Latin species name, followed by “_predicted gene ID” except for the proteins from *Volvariella volvacea*, which were labeled with “abbreviated Latin species name_protein name”. The following abbreviations were used: Sccom for *Schizophyllum commune*; Labic for *Laccaria bicolor*; Cocin for *Coprinopsis cinerea*; Pcchr for *Pchrysosporium chrysosporium*; and Vovol for *Volvariella volvacea.* The confidence levels of the nodes were tested by bootstrapping 1000 times; scores ≥80% were denoted.

To compare the compositions of small GTPases among mushrooms, the small GTPases from five basidiomycetes *Schizophyllum commune* H4-8 (v3.0; October 2013; JGI), *Laccaria bicolor* S238N-H82 (v1.0; March 2005; JGI), *Coprinopsiscinerea* Okayama7#130 (v1.0; July 2003; Broad) and *Phanerochaete Chrysosporium* RP78 (v2.0; February 2005; JGI), were also annotated by using the same method and chosen for the clustering analysis using the neighbor joining method. Results showed that each basidiomycete fungus contains all types of small GTPases (Figure 2; Table 2). The numbers of GTPases in the Rab family are consistent between the different basidiomycetes. The Rab numbers are also relatively greater than those of the GTPases in other families of GTPases in the basidiomycetes except for *Laccaria bicolor*.

### 2.3. Phylogenic Analysis of VvRan1

**Figure 3:**
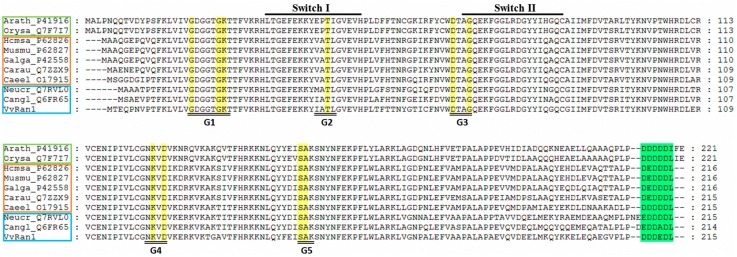
Conservation analysis of Ran amino acid sequences. Sequences were labeled with the abbreviated Latin species names followed by “_PDB ID”. The following abbreviations were used: Arath for *Arabidopsis thaliana*; Orysa for *Oryza sativa*; Homsa for *Homo sapiens*; Musmu for *Mus musculus*; Galga for *Gallus*; Carau for *Carassius auratus*; Caeel for *Caenorhabditis elegans*; Neucr for *Neurospora crassa*; and Cangl for *Candida glabrata*. The Light green box, orange box and light blue box represent the plant, animal and fungal Ran sequences, respectively. The yellow highlights represent five highly conserved G boxes among the small GTPases. The green highlights represent the acidic C-terminal sequences that are relatively conserved among animals, plants and fungi.

**Figure 4:**
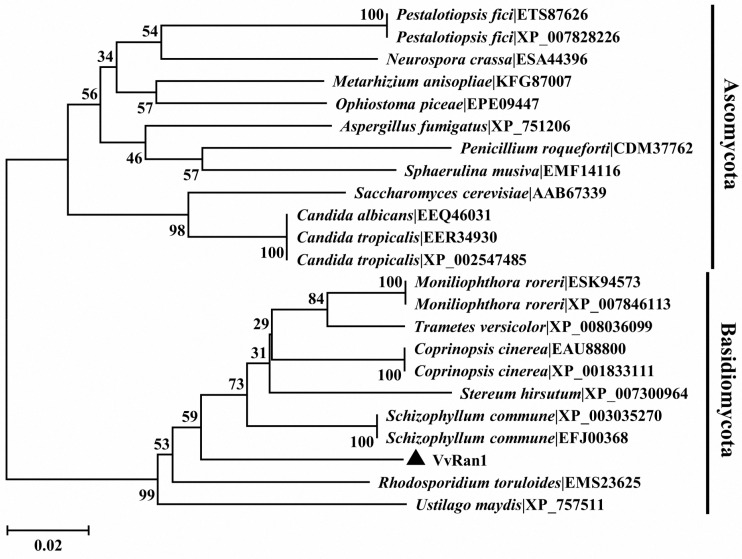
Phylogenetic analysis of *V. volvacea* and other fungal Ran sequences. All sequences except VvRan1 were downloaded from the NCBI database. The clades were named by the Latin species names, followed by the NCBI accession number. The Ran GTPase of *V. volvacea* was labeled by black triangle. The confidence levels of the nodes were tested by bootstrapping 1000 times.

The *V. volvacea* small GTPase in Ran family was termed VvRan1 in this study. To determine the relationship between VvRan1 and other Ran proteins, the amino acid sequences of VvRan1 (this study) and nine other Ran family proteins, which were downloaded from the RCSB Protein Data Bank were used for the sequence alignment. Figure 3 shows that the Ran family proteins were highly conserved in plants, animals and fungi. All of these sequences had five G box domains, two effecter regions [27] and an acidic C terminal sequence. The C-terminus of each plant Ran protein had two additional amino acid residues compared to those from animals and fungi, suggesting a close molecular evolutionary relationship between the Ran proteins of fungi and animals. To further determine the phylogenic relationship between VvRan1 and other fungal Ran GTPases, the protein sequences of VvRan1 and 22 Ran GTPases from 17 other fungal species were clustered using MEGA5.1 [28]. Figure 4 shows that the Ran GTPases clustered into two large clades. One clade contained only the Ran GTPases from Ascomycota, whereas the other clade contained only the Ran GTPases from Basidiomycota. In the Ascomycota clade, yeast Ran GTPases clustered together, while the Ran proteins of filamentous fungi clustered together. Compared with the Ran proteins of *Rhodosporidium toruloides* and *Ustilago maydis*, VvRan1 had a closer relationship with the Ran GTPases of Agaricales fungi. However, VvRan1 could not be clustered into the subclade of the Ran proteins of Agaricales fungi.

### 2.4. The Superoxide Anion (O_2_^−^) Signal Molecular Triggers vvran1 to Respone Stresses

**Figure 5:**
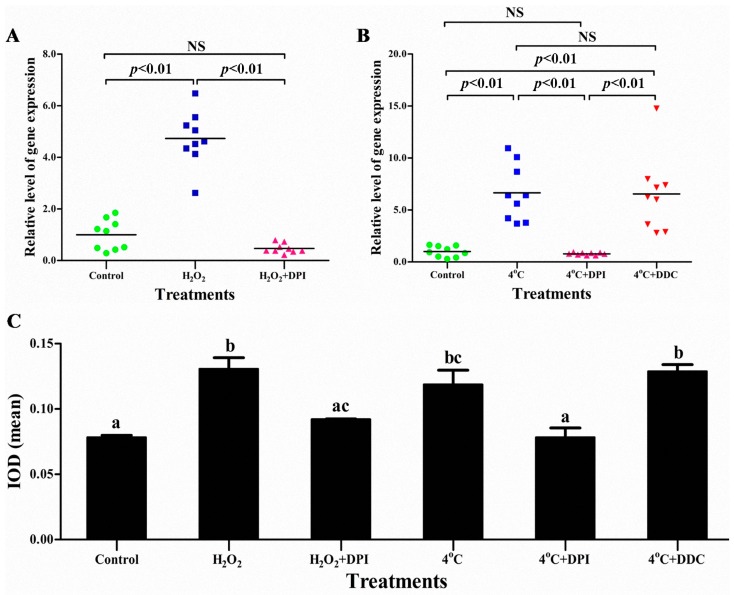
Relative expression levels of *vvran1* and intracellular superoxide anion accumulation under abiotic stresses: (**A**) relative expression levels of *vvran1* to hydrogen peroxide (H_2_O_2_) stress; (**B**) relative expression levels of *vvran1* to cold stress; and (**C**) the integrated optical density (IOD) mean of superoxide anion (O_2_^−^) detection by nitroblue tetrazolium (NBT) straining. The control, hyphae were incubated in phosphate buffered solution (PBS) buffer for 1.5 h; the H_2_O_2_ and H_2_O_2_ + DPI treatment, hyphae were incubated in PBS buffer or PBS buffer with 50 μmol/L DPI for 0.5 h, respectively, and then were switched to PBS buffer containing 10 mmol/L H_2_O_2_ and incubated for 1 h; the 4 °C and 4 °C + DPI treatment, hyphae were incubated in PBS buffer or PBS buffer with 50 μmol/L DPI for 0.5 h at 34 °C, respectively, followed by replacing the warm PBS buffer with cold PBS buffer (4 °C) and incubating the hyphae at 4 °C for 1 h; and the 4 °C + DDC treatment, hyphae were incubated for 0.5 h in PBS buffer at 34 °C, followed by exchanging the warm PBS buffer with cold PBS buffer (4 °C) containing DDC and incubating the hyphae at 4 °C for 1 h. Relative expression levels of *vvran1* in (**A**,**B**) were calculated relative to the transcript level of *vvran1* in the control and three independent experiments with nine independent replicates are shown by different color of shapes. The values of IOD mean in (**C**) are the means ± standard deviation (*n* = 3). Statistical testing of significance was performed using a one-way ANOVA and a Bonferroni’s posttest, NS means no significance, and the different letters over the columns within a graph denote significant differences (*p* < 0.05).

Ran proteins are involved in the stress responses of plants [22], humans [23], and yeast [24]. To test the relationship between *vvran1* gene and stresses in *Volvariella volvacea*, the mycelia were treated with 10 mmol/L hydrogen peroxide (H_2_O_2_, as oxidative stress) or 4 °C (as cold stress) for 1 h, respectively. RT-qPCR results showed that the *vvran1* transcript levels were 4.7- and 6.6-fold increased, respectively. These results indicated that *vvran1* gene could be regulated by both oxidative and cold stresses (Figure 5A,B).

It is known that NADPH oxidase acts as the critical role in cellular stress responses [29]. It can produce the O_2_^−^ and then converted into H_2_O_2_ by superoxide dismutase (SOD), both of them can activate specific stresses signaling, also called “redox signaling” [30]. To further understand the *vvran1* gene up-regulated mechanism, an NADPH oxidase-specific inhibitor, diphenyleneiodonium chloride (DPI) was used to inactivate the NADPH oxidases [31,32]. RT-qPCR results showed that DPI pre-incubating could completely repress the expression of *vvran1* which was up-regulated by H_2_O_2_ and 4 °C stresses (Figure 5A,B). These results could be taken as evidence that redox signaling via activation of NADPH oxidase is a reason for *vvran1* up-regulated.

Moreover, there are two effects that exogenous H_2_O_2_ can cause. One is to permeate through the plasma membrane into cell and activate signaling stresses directly [33], the other is to act as oxidative stress and trigger the cell response by NADPH oxidase. Because the DPI can only block the NADPH oxidase path but cannot prevent H_2_O_2_ into cellular, the positive result of DPI treatment under H_2_O_2_ stress suggested that specific signaling stress activated by O_2_^−^ should be the reason for *vvran1* up-regulated (Figure 5A). To further confirm this mechanism, Diethy dithiocarbamate (DDC), a SOD specific inhibitor that can keep the high level of O_2_^−^ but the low level of H_2_O_2_ in the cell [34], was added to the incubation solution during cold stress. The results showed that both the intracellular O_2_^−^ concentration and the *vvran1* expression were maintain at the high level (Figure 5B,C). These results further confirmed that O_2_^−^ but not H_2_O_2_ signal mediates the *vvran1* up-regulated expression under stress.

Additionally, the Pearson correlation coefficient method suggested that the integrated optical density (IOD) mean value of nitroblue tetrazolium (NBT) straining showed in Figure 5C was correlated with *vvran1* gene expression (*r* = 0.901, *p* < 0.05), which also indicated that the transcription of *vvran1* regulated by oxidative and cold stresses may be mediated by O_2_^−^ signal molecules.

## 3. Discussion

The cluster analysis using the neighbor joining (NJ) method grouped the human and *V. volvacea* small GTPases into five well-defined families, indicating that small GTPases within the same family are highly conserved between humans and microbes. However, there were also some highly variable amino acid residues despite the conservation of certain domains, such as the G box, between different families of small GTPases. This finding is consistent with previous results [3]. Because the yeast genome is small, it contains only 29 small GTPases [16]. A previous study showed that using only yeast as the reference for classification results in some of the small GTPases in other filamentous fungi being excluded from their appropriate families [17]. Because human small GTPases have been clearly and completely annotated and classified, we used human GTPases as the references and showed that the clustering analysis based on human small GTPases is suitable for the classification of small GTPases in filamentous fungi with large genomes.

Although Ran is the most abundant small GTPase in eukaryotes and is involved in many cellular metabolic activities, such as the assembly of nuclei, the formation of the spindle and the regulation of mitosis [4,13,14,15], each basidiomycete contains only one gene encoding a Ran GTPase. In addition, each basidiomycete contains only one Cdc42, one Rac, and one mitochondrial Rho GTPase (Figure 2). The discovery of these genes will be useful for further studies. It is worth noting that Cdc42 and Rac are evolutionarily close, which might explain the similar cellular functions of these two genes [35,36,37].

Xu and Cai [38] found that in rice, low temperature stimulation could significantly up-regulate the expression of the Ran gene *OsRAN1* and that the over-expression of *OsRAN1* could effectively improve cold tolerance in rice. Our results showed that the *vvran1* gene is also sensitive to the cold stress. Therefore, the over-expression of the *vvran1* gene may be helpful in developing cold-tolerant mushrooms. Previous studies have shown that environmental stresses can activate membrane-bound NADPH oxidase to produce active ROS signaling molecules (such as O_2_^−^ and H_2_O_2_), thereby inducing relevant genes involved in protecting cells from stress [30,39]. Yan et al. [40] found that H_2_O_2_ can up-regulate the expression of Ran/TC4 in benign mammary epithelial cells but not in malignant cells, suggesting that Ran/TC4 is involved in the antioxidant response of normal cells. Recent studies have suggested that oxidative stress can not only disrupt the distribution of Ran protein in the cell, but can also regulate the Ran-related cellular signal transduction pathways [41,42]. Based on the increased expression of *vvran1* under low temperature and oxidative stresses and the high correlations between gene expression and O_2_^−^ content, we propose that *vvran1* could be one of candidate genes in the downstream of O_2_^−^ mediated pathways which was produced by NADPH oxidase after stimulated by these abiotic stresses. According to many studies in the literature [39,43,44,45,46], intracellular H_2_O_2_ is an important signal molecular that regulates gene expression in the response to environmental stresses. However, the positive result of the experiment using the DPI under oxidative stress (H_2_O_2_ stress) and the negative result of the DDC under cold stress suggested that *vvran1* may not be regulated by intracellular H_2_O_2_.

It has been reported that the expression of both the *OsRAN1* and *OsRAN2* genes in rice can be up-regulated by cold stresses [38,47]. These genes can maintain cell division and the progression of the cell cycle by promoting the formation of an intact nuclear envelope and promoting the export of intranuclear tubulin, thereby enhancing the cold tolerance of the cell [38,47]. Some other studies have suggested that the abiotic stresses such as free radical nitric oxide production and oxidative stress can mediate Ras guanine nucleotide dissociation; this decreases the levels of intracellular RanGTP and changes its cytoplasmic distribution, thereby leading to cell death [23,41,48]. Furthermore, classical nuclear protein import can be inhibited by oxidative and other forms of stress by reducing the GTP/GDP ratio in *Saccharomyces cerevisiae* [24]. Our results showed that the expression of *vvran1* could be rapidly up-regulated by cold and oxidative stresses. This may promote nucleocytoplasmic transport, thereby enhancing the ability of the cells to tolerate stress. Needless to say, more studies are needed to reveal the detailed role of the *vvran1* gene to abiotic stresses.

## 4. Materials and Methods

### 4.1. Strains

The dikaryotic strain H1521 (collection number: ACCC52633) was used in all experiments in this study. H1521 is a heterokaryon strain generated by crossing PYd15 (ACCC52631) with PYd21 (ACCC52632), two homokaryon strains with opposite mating types.

### 4.2. Genome Sequencing, Splicing and Prediction

De novo sequencing of the whole genome of PYd21 was performed on the Solexa/Illumina platform at the Shenzhen Huada Gene Research Institute (Shenzhen, China). The genome was assembled using a SOAPdenovo assembler [49]. The NCBI accession number PRJNA171553 was assigned to the genome. A total of 11,534 encoding genes and deduced amino acid sequences were obtained using GeneMark-ES (version 2.3, Atlanta, GA, USA) [50,51].

### 4.3. Annotation of Small GTPases

RAS-related protein sequences were downloaded from the RAS Oncogene Database [25] and locally compared to the 11,534 amino acid sequences using BLASTP after a standardized library was constructed. Amino acid sequences with identities ≥30% and *e*-values ≤1 × 10^−2^ were extracted using Perl scripts and submitted to Pfam for domain prediction. Amino acid sequences containing PF00071 or PF00025 and *e*-values ≤1 × 10^−10^ were defined as small GTPases.

### 4.4. Validation of Sequence Accuracy

To identify genes encoding small GTPases in *V. volvacea*, the DNA sequences of all *V. volvacea* small GTPase coding sequences along with the 1000 bp upstream and downstream sequences were used as references to map the reads in 500 bp read pools using the ZOOM software [52]. The reads were obtained from genome sequencing, and the paired end method was used to validate the accuracy of the sequences. The software parameter settings were as follows: the distance of adjacent paired reads was set at 1 to 2000 bp; the data were presented in the Illumina format; the number of allowed mismatch bases was set to 0; and other parameters were set at default. If all base pairs of a gene were covered by reads, the sequence was considered accurate; if some of the reference sequences did not have corresponding reads, the sequences were verified using Sanger sequencing [53]. All the corrected gene sequences were used as references to map against transcriptome sequence raw reads using ZOOM software, and the software parameter settings were the same with above but the number of allowed mismatch bases was set to 40 to identify the intron region, then, predicted the amino acid sequences by ORF finder online software. If the number of mapping reads was not enough for intron identify, we predicted the gene sequence again using GeneMark-ES [50].

Finally, the integrity and accuracy of the validated amino acid sequences were determined by submitting these sequences to NCBI for BLASTP analysis. The sequences that were obviously longer than the sequences in other species or the sequences that did not align to the sequences of other species were considered to be erroneously predicted, and their extra segments were removed. For sequences that lacked intact conserved domains, we used the GENSCAN (using Vertebrate as the reference species) and Augustus (with *Laccaria bicolor* as the reference species) websites to predict the alignment again [54,55].

### 4.5. Sequence Homology Comparison and Phylogenetic Tree Construction

After using the MUSCLE program to align the sequences, a neighbor–joining tree was constructed with MEGA5.1 [28]. The structures of the conserved amino acid sequences were colored using the GeneDoc software [56].

### 4.6. Preparation of Solutions and Stress Treatments

According to our previous research, 10 mmol/L H_2_O_2_ or 4 °C treated for 1 h can significantly reduce but not completely inhibit the mycelium growth of *Volvariella volvacea* strain H1521, and hyphal growth was the fastest at pH 8 condition [57].

PBS buffer (0.02 mol/L, pH 8) containing 10 mmol/L of H_2_O_2_ was used as an oxidative stress solution. DPI (Sigma, Saint Louis, MO, USA) and DDC (Sigma, Saint Louis, MO, USA) were dissolved in sterile water and diluted to 50 μmol/L and 1 mmol/L with PBS buffer (0.02 mol/L, pH 8), respectively. Solutions for cold treatments were kept at 4 °C while solutions for other treatments were kept at 34 °C before use. The temperature mentioned in this article were allowed within 0.5 °C fluctuation.

The *V. volvacea* strain H1521 was used to test the transcriptional models of *vvran1* to different stresses. The mycelia were cultured in the solid potato dextrose agar (PDA) medium with glass papers on the surfaces of the PDA plates (Φ = 6 cm) for three days of incubation at 34 °C in the dark. To expose the mycelia to different stresses, about ten microliters of sterile PBS buffer (0.02 mol/L, pH 8) was first added to each plate to completely submerge the colonies in the buffer. The plates were incubated at 34 °C for an additional 0.5 h before stress treatment. For the DPI inhibition experiments, the colonies were pre-incubated with the DPI inhibitor solution that was added to the plates instead of PBS buffer. All stress treatments (except for cold stress treatments) were conducted at 34 °C in the dark for 1 h. For the cold stress treatments, the plates were incubated at 4 °C in the dark for 1 h.

### 4.7. vvran1 Transcript Analysis

After the stress treatment, the mycelia were quickly scraped, blot-dried and stored in a −80 °C freezer. The RNA was extracted using an E.Z.N.A.™ Plant RNA kit (Omega Bio-Tek, Norcross, GA, USA). The first strands of cDNA were synthesized using TransScript^®^ All-in-One First-Strand cDNA Synthesis SuperMix for qPCR (One-Step gDNA Removal) (TransGen Biotech, Beijing, China). Real-time fluorescent quantitative PCR (RT-qPCR) was carried out using TransStart Top Green qPCR SuperMix (TransGen Biotech, Beijing, China) on a CFX96 real-time fluorescence quantitative PCR machine (Bio-Rad, Hercules, CA, USA). The level of expression of the untreated control was used as the reference for calculating the relative expression levels using the 2^−ΔΔ*C*t^ method [58]. Glyceraldehyde-3-dehydrogenase (*GAPDH*) and 18S ribosomal RNA gene (*18S rRNA*) were used as reference genes. The PCR primers are shown in Table 3.

### 4.8. Histochemical Detection of O_2_^−^

O_2_^−^ was visually detected in the mycelia of *V. volvacea* by using NBT (Amresco, Fountain Parkway Solon, OH, USA) as substrate [59]. Briefly, the mycelia on the surfaces of the PDA plates after stresses treatment were killed quickly by liquid nitrogen, after ice melting, the mycelia were incubated with 0.05 mol/L PBS (pH 7.5) containing 0.05% NBT for 2 h at the ice-bath condition. The pictures were taken by Nikon P500 digital camera with the same exposure conditions. The computer-assisted genuine color image analysis system (imagepro-plus 6.0) was used to quantify the mean of integrated optical density.

### 4.9. Statistical Analysis

The significance of gene expression and superoxide anion content among different samples were analysed using the one-way ANOVA of variance with Bonferroni’s multiple comparisons test, and the analysis was performed by GraphPad Prism version 5.01 (San Diego, CA, USA). The correlations of gene expression patterns and O_2_^−^ contents were analyzed using the Pearson correlation coefficient method by SPSS Statistics v20 software with two-tailed test.

## Figures and Tables

**Table 1:** The sequences information of 44 Small GTPase of *Volvariella volvacea*.

Name	Gene ID_Predicted Method	GenBank ID	Name	Gene ID_Predicted Method	GenBank ID
VvArf1	GME959_T	KU882137	VvRab4	GME5663_g	KU900094
VvArf2	GME2124_T	KU882138	VvRab5	GME5664_g	KX009779
VvArf3	GME4741_g	KU882139	VvRab6	GME7647_g	KU900095
VvArf4	GME4841_g	KU882140	VvRab7	GME7649_g	KU900096
VvArf5	GME5296_g	KU882141	VvRab8	GME7988_g	KU900097
VvArf6	GME8402_T	KU882142	VvRab9	GME8752_g	KU900098
VvArf7	GME8740_g	KU882143	VvRab10	GME9500_g	KU900099
VvArf8	GME10621_g	KU882144	VvRab11	GME10910_g	KU900100
VvArf9	GME10622_g	KU882145	VvRab12	GME11319_T	KU900101
VvRho1	GME749_g	KU900090	VvRab13	GME11465_g	KU900102
VvRho2	GME1391_g	KU900105	VvRab14	GME11526_g	KU900103
VvMitRho	GME1938_g	KU900106	VvRab15	GME11774_T	KU900104
VvRho4	GME3984_G	KU900107	VvRas1	GME267_T	KX009781
VvRho5	GME4319_g	KU900108	VvRas2	GME5033_g	KU900114
VvCdc42	GME7713_T	KU900109	VvRas3	GME8078_g	KX009780
VvRho7	GME7714_g	KU900110	VvRas4	GME8593_A	KU900118
VvRho8	GME9067_g	KU900111	VvRas5	GME10486_g	KU900115
VvRho9	GME9847_g	KU900112	VvRas6	GME11128_g	KU900116
VvRac	GME11424_T	KU900113	VvRas7	GME11133_T	KX009782
VvRab1	GME3051_g	KU900091	VvRas8	GME11134_g	KU900117
VvRab2	GME5340_g	KU900092	VvRas9	GME11562_T	AHA61595
VvRab3	GME5662_g	KU900093	VvRan1	GME5409_T	KU882146

“_G” represent for GENSCAN Prediction; “_g” represent for GeneMark-ES Prediction; “_A” represent for Augustus Prediction; “_T” means the gene intron and exon regions were confirmed by transcriptom data, and the amino acid sequences were identified by ORF finder software.

**Table 2:** Distribution of five small GTPases in the Basidiomycete family.

Species	Number of Proteins						
	Arf	Rho	Ras	Rab	Ran	Total	Reference Number
*Schizophyllum commune*	7	7	5	14	1	34	34 ^a^
*Laccaria bicolor*	9	21	12	10	1	53	55 ^b^
*Pchrysosporium chrysosporium*	8	7	4	14	1	34	27 ^b^
*Coprinopsis cinerea*	8	11	5	14	1	39	29 ^b^
*Volvariella volvacea*	9	10	9	15	1	44	This study

^a^ represents the number of proteins containing PF00025 (Arf family) or PF00071 (Ras, Rho, Rab and Ran families) as reported by Raudaskoski et al. [18]; ^b^ represents the number of proteins containing PF00071 (Ras, Rho, Rab and Ran families) as reported by Rajashekar et al. [17].

**Table 3:** Primers used in real-time quantitative PCR.

Primer	Sequence (5′-3′)
*Ran1-F*	AGTTCGTCGCTGCTCCTGCTCT
*Ran1-R*	ACCCTCAGCCTGTTCCAGTTCCTT
*GAPDH-F*	CATCTTCCACTGGTGCGGCTAAG
*GAPDH-R*	GGCTTCTCAAGGCGAACGACAA
*18S rRNA-F*	TCTTGTGAAACTCTGTCGTGCTGGG
*18S rRNA-R*	TTGCCCACACCCCAAAGCTAATTCG
